# Socio‐economic gradients in prevalent tuberculosis in Zambia and the Western Cape of South Africa

**DOI:** 10.1111/tmi.13038

**Published:** 2018-03-24

**Authors:** Tom A. Yates, Helen Ayles, Finbarr P. Leacy, A. Schaap, Delia Boccia, Nulda Beyers, Peter Godfrey‐Faussett, Sian Floyd

**Affiliations:** ^1^ Institute for Global Health University College London London UK; ^2^ ZAMBART School of Medicine University of Zambia Lusaka Zambia; ^3^ Department of Clinical Research London School of Hygiene & Tropical Medicine London UK; ^4^ Data Science Centre Royal College of Surgeons in Ireland Dublin Ireland; ^5^ Department of Infectious Disease Epidemiology London School of Hygiene and Tropical Medicine London UK; ^6^ Desmond Tutu TB Centre Department of Paediatrics and Child Health Faculty of Medicine and Health Sciences Stellenbosch University South Africa

**Keywords:** tuberculosis, social epidemiology, HIV, Zambia, South Africa, tuberculose, épidémiologie sociale, VIH, Zambie, Afrique du Sud

## Abstract

**Objective:**

To describe the associations between socio‐economic position and prevalent tuberculosis in the 2010 ZAMSTAR Tuberculosis Prevalence Survey, one of the first large tuberculosis prevalence surveys in Southern Africa in the HIV era.

**Methods:**

The main analyses used data on 34 446 individuals in Zambia and 30 017 individuals in South Africa with evaluable tuberculosis culture results. Logistic regression was used to estimate adjusted odds ratios for prevalent TB by two measures of socio‐economic position: household wealth, derived from data on assets using principal components analysis, and individual educational attainment. Mediation analysis was used to evaluate potential mechanisms for the observed social gradients.

**Results:**

The quartile with highest household wealth index in Zambia and South Africa had, respectively, 0.55 (95% CI 0.33–0.92) times and 0.70 (95% CI 0.54–0.93) times the adjusted odds of prevalent TB of the bottom quartile. College or university‐educated individuals in Zambia and South Africa had, respectively, 0.25 (95% CI 0.12–0.54) and 0.42 (95% CI 0.25–0.70) times the adjusted odds of prevalent TB of individuals who had received only primary education. We found little evidence that these associations were mediated via several key proximal risk factors for TB, including HIV status.

**Conclusion:**

These data suggest that social determinants of TB remain important even in the context of generalised HIV epidemics.

## Abstract (fr)

**Objectif:**

Présenter les résultats de l'enquête ZAMSTAR 2010 sur la prévalence de la tuberculose (TB), l'une des premières grandes enquêtes sur la prévalence de la TB en Afrique australe à l’ère du VIH, selon la situation socioéconomique.

**Méthodes:**

Les principales analyses ont utilisé des données sur 34.446 individus en Zambie et 30.017 individus en Afrique du Sud avec des résultats de culture de la TB évaluables. La régression logistique a été utilisée pour estimer les rapports de cotes ajustés pour la TB prévalente selon deux mesures de la position socioéconomique: la richesse des ménages (dérivée des données sur les actifs en utilisant l'analyse des composantes principales) et le niveau d’éducation des individus. L'analyse de médiation a été utilisée pour évaluer les mécanismes potentiels des gradients sociaux observés.

**Résultats:**

Le quartile avec l'indice de richesse des ménages le plus élevé en Zambie et en Afrique du Sud avait respectivement 0.55 (IC 95%: 0.33–0.92) et 0.70 (IC 95%: 0.54–0.93) fois les probabilités ajustées de TB prévalente du quartile le plus bas. Les personnes ayant fait des études secondaires ou universitaires en Zambie et en Afrique du Sud avaient respectivement 0.25 (IC95%: 0.12–0.54) et 0.42 (IC 95%: 0.25–0.70) fois la probabilité de TB prévalente des personnes n'ayant reçu qu'une éducation primaire. Nous avons trouvé peu d’évidence que ces associations aient été médiées par plusieurs facteurs de risque clés proximaux pour la TB, y compris le statut VIH.

**Conclusion:**

Ces données suggèrent que les déterminants sociaux de la TB restent importants même dans le contexte des épidémies généralisées du VIH.

## Introduction

Socio‐economic gradients in access to health care mean the association between tuberculosis (TB) diagnosis and socio‐economic position (SEP) may not reflect social gradients in communities 1, 2, 3. Prevalence surveys enable more accurate estimation of associations between SEP and TB.

Few prevalence surveys 1, 2, 3, 4, 5, 6, 7, 8, 9 have quantified the association between SEP and prevalent TB. Four 4, 6, 8, 9 occurred in areas with generalised HIV epidemics. Of two surveys in Southern Africa 4, 8, one had substantial missing data on SEP 4. There is a study of Malawian patients detected using ‘enhanced passive case finding’^10^. Pilot surveys in ZAMSTAR communities also reported associations between SEP and prevalent TB 11, 12. The mixed findings of these studies are reviewed in the discussion.

ZAMSTAR 13, 14, 15 was a large community‐randomised trial in Zambia and the Western Cape of South Africa. Using a 2 × 2 factorial design, it tested case‐finding interventions, one delivered in the community, one in households. In 2010, after these interventions, a TB prevalence survey was conducted. Data were captured concurrently on SEP, socio‐demographic characteristics, proximal risk factors for TB, plus current TB and HIV treatment. HIV testing was offered. The household (but not the community) intervention may have reduced TB prevalence (adjusted prevalence ratio 0.82; 95% CI 0.64–1.04)^15^.

Here, we report associations between both individual educational attainment and household wealth, two measures of SEP 16, and prevalent TB in ZAMSTAR. We calculate population attributable fractions (PAFs) for SEP by each measure. We use mediation analysis 17 to evaluate potential mechanisms for social gradients and describe differences in social gradients between diagnosed and prevalent disease.

## Methods

### Ethics

The protocol was approved by ethics committees at Stellenbosch University, the University of Zambia and London School of Hygiene and Tropical Medicine. Prevalence survey participants provided written informed consent.

### Population

The survey was conducted in 16 communities in Zambia and eight in the Western Cape of South Africa. The communities, both urban and rural, had TB notification rates >400 per 100 000 per annum, high HIV prevalence and were the catchment populations of clinics offering TB diagnostics.

In each community, standard enumeration areas (SEA) were identified from census maps and visited in random order. Once 4000 adults were enrolled in a community, no further SEAs were included; for each SEA included, all households were visited. Up to three visits were made to each household.

### Measurement

Data on household‐level exposures were obtained from a responsible adult. Other data were obtained from individuals.

Participants were asked whether they had ever tested for HIV and, if so, whether they were willing to report their status. All were offered point‐of‐care HIV testing, regardless of self‐reported status. Blood sugar measurement was offered concurrently. These tests were performed in households in Zambia and at mobile centres in South Africa.

Measures of household crowding, exposure to indoor air pollution and migration were derived from answers to other questions (Table 1). The exact wording of these questions is detailed in Appendix 1.

**Table 1:** Derived binary variables used in the mediation analysis

Putative mediating factor	Variable used
HIV Status^a^	HIV positive = [HIV test positive] OR [(if HIV test not done) self‐reported HIV positive]
Household crowding	[Number of occupants, including children]/number of sleeping rooms Crowded = 3 or more people per sleeping room
IAP	Pollution if: [household mainly heated using wood or charcoal] OR [(if cooking mostly undertaken inside main house AND not using stove with combustion chamber) mainly wood or charcoal used as fuel for cooking]
Smoking	Ever smokers (either current or ex‐smokers) compared with never smokers
Malnutrition	Yes = ‘Household relied on reducing the number of meals or food in‐take in the last 18 months.’
Diabetes	Yes = [Random blood glucose ≥11.1 mmol/L] OR [self‐reported diabetes]
Alcohol consumption	Ever consumed alcohol (daily, occasional or ex‐drinkers) compared with never consumed alcohol
Migration	Years lived outside community = [Current age in years] − [‘Years lived in community’] Yes = Ever lived outside community

IAP, indoor air pollution.

a For 16% of individuals in Zambia and 39% in SA, there was no information from either serology or self‐report. This was because these individuals did not give a blood sample for HIV testing, and either reported they had never tested for HIV or that they had tested but did not know/did not wish to disclose the result of their last HIV test.

A single respiratory specimen was collected from each participant and cultured in duplicate in liquid culture. When exploring the association between SEP and prevalent TB and in the mediation analysis, we included only individuals with an ‘evaluable’ sputum sample. This meant a non‐contaminated sample which passed quality controls 15. For the main analyses, prevalent TB was defined as culture positivity.

### Conceptual framework

**Figure 1:**
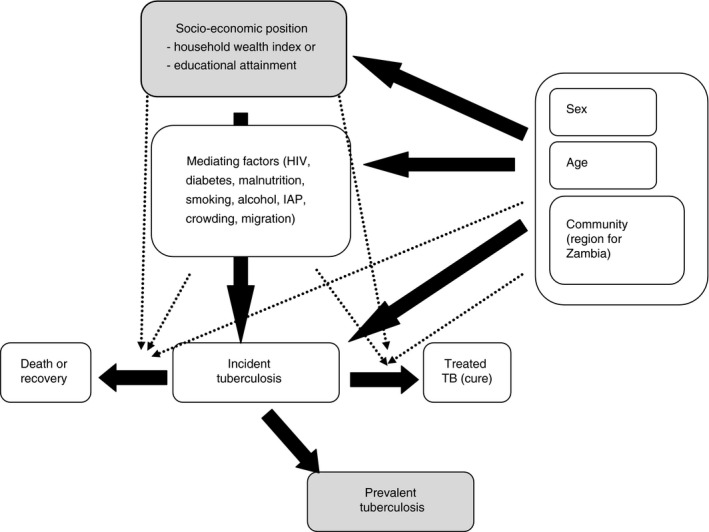
Conceptual framework.

Proximal determinants of TB infection or progression from infection to disease were considered potential mediators of the association between SEP and prevalent tuberculosis (Figure 1). Age, sex and community were considered potential confounding variables. No adjustment for previous TB was made as – given it may be similarly associated with SEP – this might artificially diminish any association between prevalent TB and SEP. To assess for social gradients in access to TB treatment, the primary analysis was repeated with self‐report of current TB treatment as the outcome.

### Analysis plan

Given their different socio‐economic landscapes, separate analyses were conducted for each country.

Household wealth indices were generated for each country by principal components analysis 18 (PCA) using data from all consented participants, irrespective of whether their sputum sample was evaluable. The variables included were household ownership of a set of assets, dwelling type, the material used to construct the floor, available sanitation facilities and the household's source of drinking water. We considered the first principal component only, with scores calculated as the sum of the factor weights for each variable. Individuals were assigned to wealth index quartiles.

Analyses of TB prevalence and current TB treatment were restricted to individuals with an evaluable sputum sample. Logistic regression models were fitted, adjusting for age group and gender, allowing the pattern by age to differ by gender.

To control for confounding by community, community was included as a fixed effect. In Zambia, communities were aggregated into four ‘regions’, each containing four communities because, in eight communities, fewer than 10 cases of prevalent TB were found. Aggregation considered force of TB infection (high or low), from a baseline survey 19, then divided communities into rural, urban (non‐Lusaka) and urban (Lusaka). Communities in the same ‘region’ were not necessarily geographically close. We accounted for clustering by SEA using robust standard errors.

PAFs were calculated for each measure of SEP in each country. We estimated the prevalent TB that would be avoided if all individuals had the same prevalence as those in the highest household wealth quartile. We then estimated the prevalent TB that would be avoided if individuals with no upper secondary education had the same prevalence as individuals with some upper secondary education, leaving the prevalence in college and university‐educated individuals unchanged.

We used the approach of Valeri and VanderWeele 17 to assess how much of the association between SEP and prevalent TB might be mediated via each of a set of proximal risk factors (Table 1). This permits decomposition of total effects into that explained by (indirect effect) and that not explained by the putative mediator (direct effect). Age, gender and community or region were held constant and clustering by SEA disregarded (in earlier analyses, it had minimal impact upon standard errors).

### Missing data

21 843 individuals in Zambia and 9793 in South Africa had complete data for all variables used in these analyses. There were no missing data on educational attainment or household wealth, meaning the main analyses excluded only 401 (Zambia) and 19 individuals (South Africa) with missing age data.

For the mediation analyses, we excluded individuals, with missing data on age, migration or household crowding – 2410 in Zambia and 961 in South Africa. A composite measure of diabetes, incorporating self‐report, was used to eliminate missingness in this variable (Table 1).

For missing HIV status, we explored two approaches. First, we reduced missingness by generating a measure incorporating self‐reported status (Table 1) then performed mediation analysis excluding those still having missing HIV status. In the second approach, we repeated the HIV mediation analysis imputing missing HIV test results assuming missing at random (MAR), i.e. that the value of the missing data, after accounting for measured predictors of HIV status, was not predicted by unobserved data. The imputation model included data on self‐reported HIV status and all variables included in the analytical model or thought to predict either HIV status or missingness of HIV status 20.

### Tools

Most analysis was conducted in Stata 13. The mediation analysis using HIV status imputed under MAR was performed in R.

### Sensitivity analyses

We repeated our main analyses stratified by gender; excluding individuals who reported previous TB; using a simple asset count as the measure of household wealth; and, for Zambia, adjusting for community rather than region as a fixed effect. We also repeated the PCA and the main analysis in Zambia using only data from the 12 urban communities.

We also repeated our main analyses, stratifying individuals who tested and/or self‐reported HIV‐positive according to whether they self‐reported that they were on anti‐retroviral therapy (ART), as follows: those who self‐reported they were HIV‐positive and that they were taking ART; those who self‐reported that they were HIV‐positive and that they were not taking ART; and those who tested or self‐reported HIV positive, but for whom, we had no data about whether they were taking ART. The latter group included people who self‐reported that they had never previously tested, self‐reported that the last time they tested the result was HIV‐negative, or declined to discuss prior HIV testing.

We restricted the diabetes and HIV mediation analyses to individuals with test results from a blood sample. Given early symptoms might alter tobacco and alcohol intake, we repeated these mediation analyses including only current vs. never smokers and heavy vs. never drinkers. We repeated the mediation analysis for malnutrition using a different measure of food security. That question asked ‘During the past 3 months, did it happen even once that you or any member of your family experienced hunger because you did not have any food to eat?’ For key mediators, HIV and IAP, we tested the sensitivity of our mediation analyses to choices made regards the level at which to fix age group, gender and community/region.

## Results

### Survey participation

There were 57 809 individuals in Zambia and 32 792 in South Africa who consented to participate in the study, representing 71% and 78% of those approached. Men were under‐represented in both countries.

There were 34 446 evaluable culture results in Zambia and 30 017 in South Africa meaning outcome data were available for 60% and 92% of individuals who consented. The proportion of unevaluable culture results differed by community. Within communities, there was little association between whether cultures were evaluable and individual characteristics. Characteristics of individuals included in the primary analysis are presented in Table 2.

**Table 2:** Baseline characteristics for each community (region in Zambia)

		Zambian regions, number (column %)				South African communities, number (column %)							
		Rural (low TST)	Other urban (low TST)	Other urban (high TST)	Lusaka (high TST)	SA1	SA2	SA3	SA4	SA5	SA6	SA7	SA8
Sex	Male	3615 (36)	2638 (35)	2354 (35)	3031 (30)	1238 (35)	1229 (32)	1395 (38)	1540 (41)	1476 (38)	1415 (37)	1518 (40)	1486 (39)
	Female	6380 (64)	4812 (65)	4377 (65)	7239 (70)	2314 (65)	2555 (68)	2288 (62)	2198 (59)	2399 (62)	2400 (63)	2244 (60)	2322 (61)
Age in years^a^	18–24	3339 (33)	2645 (36)	2395 (36)	3790 (37)	1022 (29)	975 (26)	1057 (29)	1047 (28)	1121 (29)	1400 (37)	1127 (30)	1070 (28)
	25–29	1536 (15)	1273 (17)	1135 (17)	1890 (18)	514 (14)	564 (15)	554 (15)	727 (19)	653 (17)	779 (20)	745 (20)	689 (18)
	30–34	1186 (12)	979 (13)	807 (12)	1323 (13)	383 (11)	446 (12)	454 (12)	545 (15)	492 (13)	526 (14)	604 (16)	492 (13)
	35–39	926 (9)	654 (9)	569 (8)	857 (8)	415 (12)	378 (10)	314 (8)	413 (11)	408 (11)	341 (9)	500 (13)	392 (10)
	40–49	1182 (12)	844 (11)	763 (11)	1066 (10)	659 (19)	569 (15)	502 (14)	583 (16)	635 (16)	414 (11)	456 (12)	546 (14)
	50+	1714 (17)	977 (13)	972 (14)	1223 (12)	556 (16)	851 (22)	800 (22)	420 (11)	565 (15)	353 (9)	328 (8)	614 (16)
Reported HIV status b	Positive	659 (7)	611 (8)	475 (7)	754 (7)	192 (5)	275 (7)	209 (6)	159 (4)	297 (8)	151 (4)	215 (6)	326 (9)
	Negative	4533 (45)	3218 (43)	3408 (51)	4918 (48)	1407 (40)	1639 (43)	1442 (39)	935 (25)	1584 (41)	1112 (29)	1499 (40)	1434 (38)
	Never tested	4475 (45)	3305 (44)	2677 (40)	4384 (43)	1857 (52)	1573 (42)	1865 (51)	1913 (51)	1855 (48)	2123 (56)	1461 (39)	1888 (50)
	Refuse to say	328 (3)	316 (4)	171 (3)	214 (2)	96 (3)	297 (8)	167 (5)	731 (20)	139 (4)	429 (11)	587 (16)	160 (4)
HIV test c	Positive	832 (13)	1015 (22)	793 (18)	1374 (17)	291 (14)	415 (19)	132 (16)	263 (18)	176 (19)	23 (14)	188 (19)	302 (21)
	Negative	5714 (87)	3567 (78)	3640 (82)	6545 (83)	1843 (86)	1810 (81)	698 (84)	1179 (82)	768 (81)	144 (86)	821 (81)	1112 (79)
HIV status (derived binary variable) d	Positive	1168 (14)	1242 (21)	976 (17)	1588 (17)	363 (13)	512 (17)	290 (14)	376 (18)	393 (17)	172 (13)	359 (16)	500 (20)
	Negative	7110 (86)	4624 (79)	4747 (83)	7535 (83)	2364 (87)	2468 (83)	1811 (86)	1762 (82)	1918 (83)	1190 (87)	1936 (84)	2000 (80)
Household wealth index	Very low	4311 (43)	1275 (17)	1070 (16)	1546 (15)	57 (2)	638 (17)	317 (9)	940 (25)	232 (6)	2110 (55)	854 (23)	2472 (65)
	Low	2851 (29)	1469 (20)	1452 (22)	4812 (47)	479 (13)	1473 (39)	1133 (31)	1504 (40)	565 (15)	775 (20)	966 (25)	521 (14)
	Medium	1805 (18)	2014 (27)	1699 (25)	3143 (31)	949 (27)	881 (23)	1399 (38)	938 (25)	856 (22)	459 (12)	1577 (42)	391 (10)
	High	1028 (10)	2692 (36)	2510 (37)	769 (7)	2067 (58)	792 (21)	834 (23)	356 (10)	2222 (57)	471 (13)	365 (10)	424 (11)
Education completed	None	589 (6)	354 (5)	305 (5)	723 (7)	81 (2)	186 (5)	71 (2)	209 (5)	92 (2)	110 (3)	71 (2)	319 (8)
	Primary	3392 (34)	1797 (24)	2055 (31)	3810 (37)	802 (22)	756 (20)	746 (20)	851 (23)	614 (16)	614 (16)	682 (18)	646 (17)
	Lower secondary	2478 (25)	1918 (26)	1711 (25)	2668 (26)	731 (21)	669 (18)	625 (17)	775 (21)	608 (16)	809 (21)	559 (15)	694 (18)
	Upper secondary	2793 (28)	2459 (33)	1976 (29)	2484 (24)	1808 (51)	1941 (51)	2096 (57)	1768 (47)	2346 (60)	2121 (56)	2324 (62)	2011 (53)
	College or University	743 (7)	922 (12)	684 (10)	585 (6)	130 (4)	232 (6)	145 (4)	135 (4)	215 (6)	161 (4)	126 (3)	138 (4)
Prevalent TB	Yes	39 (0.4)	50 (0.7)	34 (0.5)	69 (0.7)	79 (2.2)	87 (2.3)	92 (2.5)	116 (3.1)	108 (2.8)	73 (1.9)	56 (1.5)	91 (2.4)
	No	9956 (99.6)	7400 (99.3)	6697 (99.5)	10201 (99.3)	3473 (97.8)	3697 (97.7)	3591 (97.5)	3622 (96.9)	3767 (97.2)	3742 (98.1)	3706 (98.5)	3717 (97.6)
On TB treatment	Yes	39 (0.4)	57 (0.8)	40 (0.6)	86 (0.8)	32 (0.9)	71 (1.8)	33 (0.9)	37 (1.0)	51 (1.3)	36 (0.9)	17 (0.5)	23 (0.6)
	No	9956 (99.6)	7393 (99.2)	6691 (99.4)	10184 (99.2)	3520 (99.1)	3713 (98.1)	3650 (99.1)	3701 (99.0)	3824 (98.7)	3779 (99.1)	3745 (99.5)	3785 (99.4)
Population		9995	7450	6731	10270	3552	3784	3683	3738	3875	3815	3762	3808

TST, Tuberculin skin test.

a 401 missing observations for age in Zambia and 19 missing observations for age in South Africa.

b There was no missing data in this variable.

c HIV test result was missing in 32% in Zambia and in 66% in South Africa, respectively, because these individuals did not consent to give a blood sample for HIV testing.

d A derived variable incorporating test result plus self‐report (see Table 1).

### Wealth index

The PCA factor scores used in the household asset index are detailed in Table S1. The first principal component 18 explained 17.2% and 20.1% of total variation in Zambia and South Africa. The distributions of wealth score by country and by community or region are shown in Figures S1–S2. There was little evidence of clumping or significant truncation. Examination of household assets associated with high and low wealth scores suggested the circumstances of individuals in these households were qualitatively different. Household wealth scores correlated closely with individual educational attainment, particularly in Zambia (Figures S3–S4).

### Primary analyses

We observed associations between low SEP and prevalent TB in both countries, by both measures (Tables 3, 4). People in the quartile with the highest household wealth score in Zambia and South Africa had, respectively, 0.55 (95% CI 0.33–0.92) times and 0.70 (95% CI 0.54–0.93) times the adjusted odds of prevalent TB of those in the bottom quartile. College or university‐educated individuals in Zambia and South Africa had, respectively, 0.25 (95% CI 0.12–0.54) and 0.42 (95% CI 0.25–0.70) times the adjusted odds of prevalent TB of people with only primary education.

**Table 3:** Minimally adjusted associations of age, sex, household wealth index, educational attainment, and region or community with prevalent TB

		Number of individuals	Number with prevalent TB (%)	Odds ratio for prevalent TB (95% CI) adjusted for region/community only^a^, ^b^	*P*‐value
Zambia					
All		34 446	192 (0.6)		
Sex	Male	11 638	92 (0.8)	Referent	<0.0001
	Female	22 808	100 (0.4)	0.54 (0.42–0.70)	
Age in years among men^c^	18–24	4324	14 (0.3)	Referent	<0.0001
	25–29	1669	20 (1.2)	3.69 (1.80–7.55)	
	30–34	1365	25 (1.8)	5.72 (2.95–11.1)	
	35–39	1060	10 (0.9)	2.98 (1.32–6.73)	
	40–49	1306	12 (0.9)	2.91 (1.30–6.51)	
	50+	1880	11 (0.6)	1.87 (0.87–3.99)	
Age in years among women^c^	18–24	7845	31 (0.4)	Referent	
	25‐29	4165	25 (0.6)	1.52 (0.91–2.55)	
	30‐34	2930	12 (0.4)	1.04 (0.53–2.02)	
	35‐39	1946	14 (0.7)	1.86 (0.96–3.58)	
	40‐49	2549	15 (0.6)	1.52 (0.82–2.81)	
	50+	3006	3 (0.1)	0.26 (0.08–0.88)	
Household wealth index	Very low	8202	56 (0.7)	Referent	0.01
	Low	10 584	67 (0.6)	0.80 (0.55–1.16)	
	Medium	8661	38 (0.4)	0.53 (0.34–0.83)	
	High	6999	31 (0.4)	0.53 (0.32–0.88)	
Education completed	None	1971	12 (0.6)	0.88 (0.49–1.59)	0.04
	Primary	11 054	75 (0.7)	Referent	
	Lower secondary	8775	50 (0.6)	0.83 (0.58–1.18)	
	Upper Secondary	9712	48 (0.5)	0.72 (0.50–1.03)	
	College or University	2934	7 (0.2)	0.34 (0.16–0.72)	
Region	Rural (low TST)	9995	39 (0.4)	Referent	0.02
	Other urban (low TST)	7450	50 (0.7)	1.72 (1.16–2.56)	
	Other urban (high TST)	6731	34 (0.5)	1.30 (0.78–2.15)	
	Lusaka (high TST)	10 270	69 (0.7)	1.73 (1.14–2.62)	
South Africa					
All		30 017	702 (2.3)		
Sex	Male	11 297	333 (2.9)	Referent	<0.0001
	Female	18 720	369 (2.0)	0.66 (0.57–0.77)	
Age in years among men^d^	18–24	3379	64 (1.9)	Referent	0.006
	25–29	1928	40 (2.1)	1.10 (0.73–1.64)	
	30–34	1510	45 (3.0)	1.60 (1.15–2.22)	
	35–39	1244	45 (3.6)	1.98 (1.31–3.01)	
	40–49	1650	76 (4.6)	2.47 (1.69–3.62)	
	50+	1578	62 (3.9)	2.08 (1.47–2.94)	
Age in years among women^d^	18–24	5440	105 (1.9)	Referent	
	25–29	3297	75 (2.3)	1.18 (0.84–1.66)	
	30–34	2432	37 (1.5)	0.78 (0.53–1.16)	
	35–39	1917	29 (1.5)	0.77 (0.53–1.13)	
	40–49	2714	50 (1.8)	0.92 (0.65–1.32)	
	50+	2909	73 (2.5)	1.27 (0.87–1.85)	
Household wealth index	Very low	7620	196 (2.6)	Referent	0.12
	Low	7416	175 (2.4)	0.82 (0.63–1.06)	
	Medium	7450	166 (2.2)	0.79 (0.60–1.04)	
	High	7531	165 (2.2)	0.71 (0.54–0.94)	
Education completed	None	1139	40 (3.5)	1.04 (0.70–1.53)	<0.0001
	Primary	5711	189 (3.3)	Referent	
	Lower secondary	5470	137 (2.5)	0.75 (0.62–0.91)	
	Upper Secondary	16 415	319 (1.9)	0.59 (0.49–0.71)	
	College or University	1282	17 (1.3)	0.39 (0.23–0.65)	
Community	SA1	3552	79 (2.2)	Referent	0.0001
	SA2	3784	87 (2.3)	1.03 (0.68–1.58)	
	SA3	3683	92 (2.5)	1.13 (0.78–1.64)	
	SA4	3738	116 (3.1)	1.41 (0.99–1.99)	
	SA5	3875	108 (2.8)	1.26 (0.87–1.83)	
	SA6	3815	73 (1.9)	0.86 (0.58–1.27)	
	SA7	3762	56 (1.5)	0.66 (0.46–0.96)	
	SA8	3808	91 (2.4)	1.08 (0.73–1.60)	

a Clustering by SEA accounted for using Robust Standard Errors.

b Note, in both countries, adjusting for region/community had very little impact on odds ratios and their standard errors.

c 401 observations for age missing in Zambia.

d 19 observations for age missing in South Africa.

**Table 4:** Adjusted odds ratios (aOR) for prevalent TB and for being on TB treatment by measures of socio‐economic position for each country

		aOR for prevalent TB (95% CI)^a^, ^b^	*P*‐value	aOR for being on TB treatment (95% CI)^a^, ^b^	*P*‐value
Zambia					
Household wealth index	Very low	Referent	0.03	1	0.17
	Low	0.79 (0.54–1.16)		0.88 (0.61–1.26)	
	Medium	0.54 (0.34–0.85)		0.71 (0.48–1.04)	
	High	0.55 (0.33–0.92)		0.62 (0.37–1.04)	
Education completed	None	1.39 (0.77–2.50)	0.001	0.70 (0.35–1.39)	0.07
	Primary	Referent		Referent	
	Lower Secondary	0.76 (0.52–1.10)		0.87 (0.63–1.21)	
	Upper Secondary	0.66 (0.44–0.98)		0.69 (0.46–1.03)	
	College or University	0.25 (0.12–0.54)		0.38 (0.18–0.81)	
Number of assets owned	0‐1	Referent	0.003	Referent	0.0002
	2	0.85 (0.57–1.26)		0.76 (0.53–1.09)	
	3	0.55 (0.36–0.85)		0.57 (0.39–0.83)	
	4	0.44 (0.27–0.71)		0.47 (0.32–0.69)	
	5‐8	0.62 (0.39–0.99)		0.41 (0.25–0.68)	
		aOR for prevalent TB (95% CI)^a^, ^c^	*P*‐value	aOR for being on TB treatment (95% CI)^a^, ^c^	*P*‐value
South Africa					
Household wealth index	Very low	1	0.09	1	0.0006
	Low	0.81 (0.63–1.04)		0.78 (0.52–1.18)	
	Medium	0.78 (0.60–1.02)		1.01 (0.67 1.52)	
	High	0.70 (0.54–0.93)		0.53 (0.33–0.85)	
Education completed	None	1.05 (0.71–1.55)	<0.0001	1.41 (0.80–2.48)	0.0009
	Primary	Referent		Referent	
	Lower Secondary	0.81 (0.67–0.98)		0.66 (0.45–0.95)	
	Upper Secondary	0.63 (0.52–0.77)		0.54 (0.38–0.78)	
	College or University	0.42 (0.25–0.70)		0.67 (0.35–1.27)	
Number of assets owned	0–1	Referent	0.003	Referent	0.003
	2	0.94 (0.69–1.29)		0.93 (0.56–1.55)	
	3	0.76 (0.55–1.06)		0.78 (0.46–1.32)	
	4	0.70 (0.53–0.92)		0.60 (0.39–0.92)	
	5–8	0.57 (0.41–0.80)		0.48 (0.30–0.78)	

a Adjusted for age group, gender and community or region, with clustering by SEA accounted for using robust standard errors.

b 401 observations for age missing in Zambia – these individuals not included in model.

c 19 observations for age missing in South Africa – these individuals not included in model.

### Population attributable fractions

Were everyone to have the TB prevalence of those in the highest quartile of household wealth, then 23.5% (95% CI −10.7–47.1%) and 13.5% (−0.6–25.6%) of prevalent TB might be avoided in Zambia and South Africa, respectively (Table S2). Were individuals with no upper secondary education to have the rates of TB of individuals with some upper secondary education, then 19.3% (95% CI −3.1–36.9%) and 15.1% (95% CI 7.7–21.9%) of prevalent TB might be avoided in Zambia and South Africa, respectively (Table S2).

### Mediation analyses

The associations between SEP and HIV status are shown in Table S3. The associations between SEP and putative mediators are shown in Tables S4–S5. The associations between putative mediators and prevalent TB are shown in Tables S6–S7. The adjusted odds ratios for prevalent TB, for HIV‐positive people *vs*. HIV‐negative people, were 4.25 (95% CI 3.14–5.75) and 2.76 (95% CI 2.22–3.44), respectively, in Zambia and South Africa.

There was little evidence, after accounting for covariates, that the observed associations between prevalent TB and low SEP were mediated by any proximal risk factors considered (Table 5); that is, the conditional natural indirect effects were all approximately equal to one, with conditional natural direct and indirect effects presented on the odds ratio scale 17.

**Table 5:** Mediation analysis for the association between socio‐economic position and prevalent TB

Putative mediator		Zambia^a^			South Africa^b^		
		Direct Effect Odds Ratio (95% CI)	Indirect Effect Odds Ratio (95% CI)	Total Effect Odds Ratio (95% CI)	Direct Effect Odds Ratio (95% CI)	Indirect Effect Odds Ratio (95% CI)	Total Effect Odds Ratio (95% CI)
Educational attainment							
Smoking	None	1.40 (074–2.67)	1.01 (0.96–1.06)	1.41 (0.74–2.70)	1.11 (0.78–1.58)	0.99 (0.87–1.13)	1.10 (0.75–1.61)
	Primary	Referent			Referent		
	Lower secondary	0.76 (0.52–1.10)	0.96 (0.92–0.99)	0.72 (0.50–1.06)	0.81 (0.64–1.02)	1.00 (0.92–1.07)	0.80 (0.63–1.03)
	Upper secondary	0.70 (0.47–1.03)	0.92 (0.87–0.98)	0.64 (0.44–0.95)	0.67 (0.54–0.83)	0.96 (0.89–1.03)	0.64 (0.51–0.81)
	College or university	0.29 (0.13–0.63)	0.92 (0.86–0.98)	0.26 (0.12–0.58)	0.44 (0.26–0.75)	0.89 (0.76–1.04)	0.39 (0.23–0.68)
Alcohol	None	1.45 (0.76–2.77)	0.97 (0.92–1.02)	1.41 (0.74–2.69)	1.12 (0.78–1.59)	0.99 (0.90–1.08)	1.10 (0.76–1.59)
	Primary	Referent			Referent		
	Lower secondary	0.74 (0.51–1.07)	1.00 (0.98–1.03)	0.74 (0.51–1.07)	0.81 (0.64–1.02)	0.99 (0.94–1.05)	0.80 (0.63–1.02)
	Upper secondary	0.67 (0.45–0.98)	0.99 (0.96–1.02)	0.66 (0.45–0.97)	0.65 (0.52–0.81)	0.98 (0.93–1.03)	0.64 (0.51–0.80)
	College or university	0.27 (0.12–0.59)	1.01 (0.98–1.05)	0.27 (0.12–0.59)	0.42 (0.24–0.71)	0.97 (0.89–1.06)	0.40 (0.23–0.69)
Malnutrition	None	1.37 (0.72–2.60)	1.00 (0.97–1.03)	1.37 (0.72–2.61)	1.10 (0.77–1.56)	1.00 (1.00–1.00)	1.10 (0.77–1.56)
	Primary	Referent			Referent		
	Lower secondary	0.74 (0.50–1.07)	1.00 (0.98–1.02)	0.73 (0.50–1.07)	0.80 (0.63–1.02)	1.00 (1.00–1.00)	0.80 (0.63–1.02)
	Upper secondary	0.66 (0.45–0.97)	1.00 (0.96–1.03)	0.66 (0.44–0.97)	0.64 (0.52–0.80)	1.00 (1.00–1.00)	0.64 (0.52–0.80)
	College or university	0.26 (0.12–0.58)	1.00 (0.95–1.05)	0.26 (0.12–0.57)	0.41 (0.24–0.69)	1.00 (1.00–1.00)	0.41 (0.24–0.69)
Diabetes	None	1.37 (0.72–2.61)	1.00 (1.00–1.00)	1.37 (0.72–2.61)	1.09 (0.77–1.56)	1.00 (1.00–1.00)	1.09 (0.77–1.56)
	Primary	Referent			Referent		
	Lower secondary	0.73 (0.50–1.07)	1.00 (1.00–1.00)	0.73 (0.50–1.07)	0.80 (0.64–1.02)	1.00 (1.00–1.00)	0.80 (0.64–1.02)
	Upper secondary	0.66 (0.44–0.97)	1.00 (1.00–1.00)	0.66 (0.44–0.97)	0.64 (0.52–0.80)	1.00 (1.00–1.00)	0.64 (0.52–0.80)
	College or university	0.26 (0.12–0.57)	1.00 (1.00–1.00)	0.26 (0.12–0.57)	0.40 (0.24–0.69)	1.00 (1.00–1.00)	0.40 (0.24–0.69)
Indoor air pollution	None	1.37 (0.72–2.60)	1.00 (0.89–1.12)	1.37 (0.71–2.63)	Very few South Africans exposed to indoor air pollution		
	Primary	Referent					
	Lower secondary	0.75 (0.52–1.10)	0.98 (0.93–1.05)	0.74 (0.51–1.08)			
	Upper secondary	0.69 (0.46–1.01)	0.97 (0.90–1.04)	0.66 (0.45–0.98)			
	College or university	0.29 (0.13–0.63)	0.92 (0.82–1.05)	0.26 (0.12–0.58)			
Household crowding	None	1.36 (0.72–2.59)	1.00 (0.96–1.05)	1.37 (0.72–.2.60)	1.09 (0.77–1.56)	1.00 (0.93–1.08)	1.09 (0.76–1.57)
	Primary	Referent			Referent		
	Lower secondary	0.73 (0.50–1.06)	1.00 (0.98–1.03)	0.73 (0.50–1.07)	0.80 (0.63–1.01)	1.00 (0.96–1.04)	0.80 (0.63–1.02)
	Upper secondary	0.65 (0.44–0.96)	1.01 (0.98–1.04)	0.66 (0.44–0.97)	0.64 (0.52–0.80)	1.00 (0.96–1.04)	0.64 (0.51–0.80)
	College or university	0.26 (0.12–0.56)	1.02 (0.96–1.08)	0.26 (0.12–0.57)	0.40 (0.24–0.68)	1.00 (0.93–1.09)	0.40 (0.24–0.69)
Migration	None	1.39 (0.73–2.65)	0.98 (0.83–1.16)	1.37 (0.70–2.66)	1.10 (0.77–1.57)	1.00 (0.65–1.54)	1.10 (0.63–1.93)
	Primary	Referent			Referent		
	Lower secondary	0.74 (0.51–1.08)	0.99 (0.92–1.07)	0.74 (0.50–1.08)	0.80 (0.63–1.01)	1.00 (0.83–1.20)	0.80 (0.59–1.08)
	Upper secondary	0.67 (0.46–1.00)	0.98 (0.90–1.06)	0.66 (0.44–0.98)	0.64 (0.51–0.79)	1.00 (0.85–1.18)	0.64 (0.48–0.84)
	College or university	0.27 (0.12–0.60)	0.96 (0.84–1.09)	0.26 (0.12–0.58)	0.40 (0.23–0.68)	1.00 (0.79–1.26)	0.40 (0.22–0.71)
HIV (complete case^c^)	None	1.77 (0.92–3.41)	0.99 (0.98–1.00)	1.75 (0.91–3.37)	1.47 (0.93–2.33)	0.99 (0.98–1.00)	1.46 (0.92–2.31)
	Primary	Referent			Referent		
	Lower secondary	0.86 (0.58–1.29)	1.00 (1.00–1.00)	0.87 (0.58–1.29)	1.17 (0.86–1.59)	0.99 (0.98–1.00)	1.16 (0.85–1.57)
	Upper secondary	0.81 (0.53–1.23)	0.98 (0.98–0.99)	0.80 (0.52–1.22)	1.01 (0.76–1.35)	0.98 (0.96–0.99)	0.99 (0.74–1.32)
	College or university	0.30 (0.12–0.75)	0.97 (0.96–0.99)	0.29 (0.12–0.73)	0.83 (0.46–1.51)	0.95 (0.92–0.98)	0.79 (0.44–1.43)
HIV (MAR d)	None	1.45 (0.78–2.71)	0.98 (0.97–1.00)	1.43 (0.77–2.66)	1.03 (0.71–1.48)	1.00 (0.97–1.02)	1.02 (0.71–1.47)
	Primary	Referent			Referent		
	Lower secondary	0.77 (0.54–1.12)	1.00 (1.00–1.01)	0.78 (0.54–1.12)	0.83 (0.65–1.05)	0.99 (0.97–1.00)	0.82 (0.65–1.03)
	Upper secondary	0.73 (0.49–1.07)	0.98 (0.97–0.99)	0.71 (0.49–1.05)	0.67 (0.54–0.83)	0.98 (0.97–0.99)	0.66 (0.53–0.81)
	College or university	0.30 (0.14–0.66)	0.97 (0.96–0.99)	0.29 (0.13–0.64)	0.46 (0.27–0.78)	0.95 (0.92–0.98)	0.44 (0.26–0.74)
Household wealth							
Smoking	Very low	Referent			Referent		
	Low	0.81 (0.55–1.19)	0.96 (0.93–0.99)	0.78 (0.53–1.14)	0.79 (0.63–0.99)	1.00 (0.93–1.07)	0.78 (0.62–1.00)
	Medium	0.58 (0.37–0.89)	0.93 (0.88–0.98)	0.54 (0.35–0.83)	0.76 (0.60–0.96)	0.98 (0.91–1.06)	0.75 (0.58–0.96)
	High	0.60 (0.37–0.96)	0.92 (0.86–0.98)	0.55 (0.34–0.88)	0.69 (0.53–0.88)	0.97 (0.90–1.05)	0.66 (0.51–0.87)
Alcohol	Very low	Referent			Referent		
	Low	0.80 (0.55–1.17)	0.99 (0.97–1.02)	0.79 (0.54–1.16)	0.77 (0.61–0.97)	1.02 (0.97–1.07)	0.79 (0.62–0.99)
	Medium	0.55 (0.36–0.85)	1.00 (0.97–1.02)	0.55 (0.36–0.85)	0.74 (0.59–0.94)	1.01 (0.96–1.06)	0.75 (0.59–0.96)
	High	0.56 (0.35–0.89)	1.01 (0.98–1.04)	0.56 (0.35–0.90)	0.68 (0.53–0.87)	0.98 (0.93–1.04)	0.67 (0.52–0.86)
Malnutrition	Very low	Referent			Referent		
	Low	0.79 (0.54–1.16)	1.00 (0.98–1.02)	0.79 (0.54–1.16)	0.79 (0.63–0.99)	1.00 (1.00–1.00)	0.79 (0.63–0.99)
	Medium	0.55 (0.35–0.85)	1.00 (0.96–1.04)	0.55 (0.35–0.84)	0.75 (0.59–0.95)	1.00 (1.00–1.00)	0.75 (0.59–0.95)
	High	0.56 (0.35–0.90)	1.00 (0.94–1.05)	0.56 (0.35–0.89)	0.67 (0.52–0.86)	1.00 (1.00–1.00)	0.67 (0.52–0.86)
Diabetes	Very low	Referent			Referent		
	Low	0.79 (0.54–1.15)	1.00 (1.00–1.00)	0.79 (0.54–1.15)	0.79 (0.63–0.99)	1.00 (1.00–1.00)	0.79 (0.63–0.99)
	Medium	0.54 (0.35–0.84)	1.00 (1.00–1.01)	0.54 (0.35–0.84)	0.75 (0.59–0.95)	1.00 (1.00–1.00)	0.75 (0.59–0.95)
	High	0.55 (0.34–0.89)	1.00 (1.00–1.01)	0.55 (0.34–0.89)	0.67 (0.52–0.86)	1.00 (1.00–1.00)	0.67 (0.52–0.86)
Indoor air pollution	Very low	Referent			Very few South Africans exposed to indoor air pollution		
	Low	0.80 (0.55–1.18)	0.99 (0.92–1.06)	0.80 (0.54–1.17)			
	Medium	0.59 (0.38–0.91)	0.94 (0.86–1.04)	0.55 (0.36–0.86)			
	High	0.63 (0.38–1.03)	0.89 (0.76–1.04)	0.56 (0.35–0.91)			
Household crowding	Very low	Referent			Referent		
	Low	0.79 (0.54–1.15)	1.00 (0.98–1.02)	0.79 (0.54–1.16)	0.78 (0.62–0.99)	1.00 (0.95–1.05)	0.79 (0.62–0.99)
	Medium	0.54 (0.35–0.84)	1.00 (0.98–1.03)	0.55 (0.35–0.84)	0.75 (0.59–0.95)	1.00 (0.95–1.05)	0.75 (0.59–0.96)
	High	0.55 (0.34–0.88)	1.01 (0.97–1.05)	0.55 (0.35–0.89)	0.66 (0.51–0.86)	1.00 (0.94–1.07)	0.67 (0.51–0.86)
Migration	Very low	Referent			Referent		
	Low	0.80 (0.55–1.17)	0.99 (0.91–1.07)	0.79 (0.53–1.16)	0.77 (0.61–0.97)	1.00 (0.86–1.16)	0.77 (0.59–1.02)
	Medium	0.56 (0.36–0.86)	0.98 (0.90–1.06)	0.54 (0.35–0.85)	0.74 (0.58–0.93)	1.00 (0.85–1.17)	0.74 (0.55–0.98)
	High	0.58 (0.36–0.93)	0.96 (0.88–1.06)	0.56 (0.34–0.90)	0.65 (0.50–0.84)	1.00 (0.85–1.17)	0.65 (0.48–0.88)
HIV (complete case^c^)	Very low	Referent			Referent		
	Low	0.80 (0.53–1.21)	1.00 (1.00–1.00)	0.80 (0.53–1.12)	0.72 (0.53–0.96)	0.98 (0.97–0.99)	0.70 (0.52–0.95)
	Medium	0.62 (0.39–0.99)	0.99 (0.99–1.00)	0.61 (0.39–0.98)	0.82 (0.61–1.10)	0.99 (0.98–1.00)	0.81 (0.60–1.09)
	High	0.68 (0.40–1.15)	0.98 (0.96–0.99)	0.67 (0.39–1.12)	0.66 (0.48–0.92)	0.98 (0.96–0.99)	0.65 (0.47–0.90)
HIV (MAR^d^)	Very low	Referent			Referent		
	Low	0.78 (0.54–1.13)	1.00 (1.00–1.01)	0.78 (0.54–1.14)	0.86 (0.69–1.08)	0.97 (0.96–0.99)	0.84 (0.67–1.06)
	Medium	0.57 (0.37–0.87)	0.99 (0.98–1.00)	0.57 (0.37–0.87)	0.83 (0.65–1.05)	0.98 (0.96–0.99)	0.81 (0.64–1.03)
	High	0.64 (0.40–1.02)	0.97 (0.96–0.99)	0.62 (0.39–0.99)	0.74 (0.57–0.96)	0.97 (0.94–0.99)	0.72 (0.56–0.93)

a 2410 Zambians with missing data on age, household crowding or migration excluded from these mediation analyses.

b 961 South Africans with missing data on age, household crowding or migration excluded from these mediation analyses.

c The measure incorporating self‐report (see Table 1).

d Missing HIV status imputed under the Missing at Random assumption.

### Social gradients in receipt of TB treatment

Social gradients in prevalent TB observed in Zambia were stronger than for diagnosed TB, but the trend was similar. In South Africa, the strength of the association between education and current TB treatment was similar to that between education and prevalent TB. There was also an association between wealth index and current TB treatment, but we did not observe that the odds of diagnosed TB fell with every increment in SEP, as with prevalent TB. These data are in Table 4.

### Sensitivity analyses

Our findings were robust to the sensitivity analyses undertaken. Importantly, the social gradient in prevalent TB using a simple asset count was similar to that seen using the household wealth index (Table 4).

However, there were insufficient data to permit exploration of ART use, in addition to HIV status. Among 24 440 Zambians for whom we had information on HIV status, 842 self‐reported they were HIV‐positive and not taking ART, 1611 self‐reported they were HIV‐positive and taking ART (6.6% of the total population, and 32.4% of those who tested or self‐reported HIV‐positive), and 2521 were HIV‐positive based on survey testing, but they did not self‐report they were HIV‐positive and so there was no information on ART use. Among 11 340 South Africans for whom we had information on HIV status, 767 self‐reported they were HIV‐positive and not taking ART, 1022 self‐reported they were HIV‐positive and taking ART (9.0% of the total population, and 34.5% of those who tested or self‐reported HIV‐positive), and 1176 were HIV‐positive based on survey testing, but they did not self‐report that they were HIV‐positive and so there was no information on ART use.

## Discussion

### Main findings

We observed strong social gradients in prevalent TB in two very different settings in Southern Africa. These were steeper in Zambia. These gradients were not explained by a number of putative mediating variables. The association between SEP and being on TB treatment was less clear. As observed in the Zambian ZAMSTAR pilot prevalence survey 21 (and in a study of diagnosed TB in Brazil 22), a substantial proportion of TB might be avoided if people with low SEP suffered the same burden as those with high SEP.

Poor educational attainment was more strongly associated with prevalent TB than household wealth. Individual‐level SEP may be more important than household‐level SEP; absolute measures of SEP may better predict TB than relative measures; human capital (knowledge or skills) might be more protective than wealth or living conditions; or longer‐term disadvantage might be important with education fixed early in adulthood whereas asset ownership may fluctuate. Alternatively, assets included in our index may not fully explain variation in SEP in these communities.

### Limitations

The size of the study and the consistency of our findings in two settings and by two measures of SEP suggest this is not a chance finding. However, a number of biases might affect our estimates.

Within study communities, it is possible sickness or employment affected recruitment into the study. We would expect any bias introduced to be modest.

The weighting of components of the wealth indices broadly agreed with our beliefs about which assets were associated with relative wealth. However, choices made in the construction of wealth indices can bias estimates of the association between SEP and TB.

Inclusion of assets directly associated with the outcome can lead to overestimation of the health inequalities 23. For TB, it has been argued that measures of household construction should not be included, given they may affect ventilation 24. That we obtain similar results when using a simple asset count, without measures of household construction, suggests including them did not substantially affect our estimates.

The inclusion of ‘urban’ assets in wealth indices can theoretically attenuate the association between low SEP and TB, given urban areas tend to be wealthier and have a higher burden of TB 16, 24. However, we obtained very similar results in Zambia when both the PCA and the main analysis were undertaken using only data from the 12 urban communities. A previous study in Zambia suggested excluding urban variables did not alter the association between SEP and TB 24. Additional discriminatory power obtained by including urban assets may offset any attenuation. Many people in ‘rural’ communities in this study were living in peri‐urban areas.

The association between SEP and prevalent TB did not appear to be mediated by any of the proximal risk factors measured. An important caveat here is that many of these risk factors were imperfectly measured. Our measure of diabetes was insensitive 25. We did not measure protein intake, which an earlier study from Zambia suggested might be the component of malnutrition that is associated with TB 11. We had no data on recent migration.

Three variables were dichotomised to enable them to be included in the mediation analysis. Of these, diabetes was associated with higher SEP, so could not explain the association between low SEP and prevalent TB observed. Furthermore, in these communities, the association between diabetes and prevalent TB is weak and diabetes only thought to explain around 1.0% of prevalent TB (95% CI 0.1–1.9%) 26. No association was observed between household crowding and prevalent TB, even when household crowding was more finely categorised. Time spent outside the community appeared protective, at least in Zambia, and was associated with higher SEP in Zambia and lower SEP in South Africa. However, in Zambia, the majority of people (91%) had either never lived outside the community or had done so for more than 10 years – that is, it was already essentially a binary variable. A finer categorisation of the migration variable was not informative, as there were too few cases of TB among individuals who had lived outside the community for between 1 and 10 years.

Many participants declined to test for HIV. The inclusion of self‐reported status in one measure of HIV will have resulted in some misclassification, given HIV remains stigmatised and a proportion of individuals will have become HIV‐positive since their last test. However, the odds ratios for the association between HIV and prevalent TB were consistent with previous studies, and similar in Zambia and South Africa.

There was little evidence to show that HIV mediated the association between SEP and prevalent TB. This was surprising given lower SEP was associated with HIV positivity in complete case analysis and HIV infection clearly associated with prevalent TB. This was true for both men and women and among both younger and older individuals.

In an analysis imputing missing HIV serology data assuming MAR, we also did not find evidence of mediation. However, HIV status in population based surveys is often missing not at random (MNAR) 27, 28. Individuals who know themselves to be HIV positive are more likely to decline testing. The imputation methods we used are not valid if there is substantial departure from MAR. We have explored the extent to which MNAR might affect our conclusions in a sensitivity analysis, finding little evidence of mediation by HIV status across a range of plausible MNAR assumptions 20.

The association between SEP and HIV may be both complex and dynamic 29. Given HIV is a key risk factor for TB, in settings with a stronger social gradient in HIV positivity, we would expect HIV to, at least partially, mediate any association between SEP and prevalent TB. However, improvements in HIV care, including the earlier initiation of ART, may attenuate this effect.

ART modifies the association between HIV and TB 30, but we were unable to examine the effect of ART due to data limitations. An increasing proportion of HIV‐positive people are taking ART, and with WHO now recommending ART initiation regardless of CD4 count, this trend is likely to continue. The impact of ART on the social patterning of TB should be examined in future analyses. Other potential mediators of the social gradient in prevalent TB were also not examined. These include social contact carrying a TB risk and structural barriers to accessing TB treatment.

Our analysis accounts for within‐community and not between‐community social gradients in prevalent TB. A brief exploratory analysis of the Zambian data suggested that modest between‐community social gradients also existed with higher TB prevalence observed in poorer and less well‐educated communities.

### Strength of evidence for a causal association

A key assumption behind the PAFs that we present is that the association between SEP and prevalent TB that we describe is causal.

TB disease is a cause of impoverishment 31. As educational attainment is usually fixed in early adult life, reverse causality is unlikely to explain its association with prevalent TB. As a measure of SEP, household assets are considered relatively stable to short‐term economic shocks 32, 33, such as illness. However, individuals with TB may sell assets to fund hospital visits or when they became too unwell to work. This would result in overestimates of the association between household wealth and prevalent TB. However, prevalent TB or current receipt of TB treatment was not strongly associated with household sale of assets in either country.

In Zambia, 8.2% of individuals included in the primary analysis lived in households reporting sale of assets in the preceding 18 months. The equivalent figures for individuals with prevalent TB and individuals in receipt of TB treatment were 12.5% and 11.3%, respectively. Sale of assets was reported more frequently in less asset‐rich households. In South Africa, 3.1% of individuals in the primary analysis, 3.2% of individuals with prevalent TB and 4.3% of individuals on TB treatment lived in households reporting sale of assets in the preceding 18 months.

An alternative explanation for the association between SEP and prevalent TB that we describe is residual confounding. Under our conceptual framework, proximal determinants of prevalent TB would be considered to be on the causal pathway rather than putative confounding variables. However, we cannot exclude the possibility that some of the observed association is explained by confounding by upstream factors, such as a healthier environment or better governance. Including community or region as a fixed effect might not control for such upstream factors if, for example, they were operating at a different scale or if political constituencies and study communities did not overlap. The extent to which this matters depends on whether one wishes to isolate the pure effect of SEP from its environmental and contextual determinants.

### Results in context

The social gradient in diagnosed disease was less clear than for prevalent disease. The effects were subtle, but this might suggest some social patterning in access to treatment, as noted elsewhere 1, 2, 3. Note the ZAMSTAR communities had their diagnostic capacity strengthened through participation in this trial.

A prevalence survey in Myanmar found prevalent TB was not associated with SEP after stratifying by rurality 3. The recent prevalence survey in Zambia observed prevalent TB was associated with lower SEP in urban areas but no association between prevalent TB and SEP in rural areas 8. However, our results are consistent with large TB prevalence surveys from South India 1, the Philippines 5, Vietnam 5, Bangladesh 2, 5, 34, Shandong Province in China 7, Kenya 5 and Tanzania 9 which all found prevalent TB to be associated with lower SEP. They are also consistent with a prevalence survey in Zimbabwe which found a non‐significant reduction in the odds of prevalent TB per asset owned in univariable analysis 4.

ZAMSTAR pilot prevalence surveys in Zambia 11 and the Western Cape 12 both reported associations between lower SEP and prevalent TB, with some evidence that this was mediated by poor protein intake 11.

Studies of the association between diagnosed disease and SEP in Southern Africa 10, 35, 36, 37 have yielded divergent results, perhaps due to differing social gradients in access to health care.

Odone reported interesting differences in the associations between various measures of SEP and diagnosed TB in a large study from Northern Malawi 10. Whilst ownership of assets appeared protective, better household construction and working in the cash rather than the subsistence economy were associated with higher rates of TB 10.

There are plausible reasons why aspects of higher SEP might place one at greater risk of TB infection 10, 38. Employed individuals may have greater exposure to other people whilst commuting; in the workplace; and, perhaps, via more frequent attendance at social or commercial venues, made possible by their earnings. Alternatively, better constructed homes may be less well ventilated 39. This might explain the association between better household construction and diagnosed TB observed by Odone 10. However, a growing body of evidence suggests that most *Mycobacterium tuberculosis* transmission in Southern Africa occurs outside the household 40, 41, 42, 43.

## Conclusions

We have shown that steep socio‐economic gradients in prevalent TB persist in Southern African communities with high HIV prevalence. These associations are probably causal. If so, low SEP is responsible for a substantial proportion of prevalent TB in these communities. We were unable to identify any mediating factors that explained these associations. Confirmation of the previously noted 11 association between poor protein intake and prevalent TB would be valuable. Future studies of the association between SEP and TB must consider differences by SEP in access to TB treatment as part of the explanation for any observed associations. Longitudinal studies would be valuable in establishing causality and, potentially, in measuring the effect of interventions to reduce poverty.

That previous studies from HIV‐endemic areas of Kenya 5, Zimbabwe 4, Tanzania 9, the Western Cape 12, and (at least urban) Zambia 8, 11 have also found an association between low SEP and prevalent TB suggests this may be the case more generally. These findings lend support to the inclusion of poverty alleviation and social protection as ‘key actions’ under Pillar 2 of WHO's End TB Strategy 44. National Treatment Programmes in HIV‐endemic settings, as elsewhere, must ensure that their services can be accessed by individuals with little education, members of asset‐poor households, and other less advantaged members of the community.

## Supporting information


**Figure S1**. The distribution of household wealth index overall and by community in Zambia.
**Figure S2**. The distribution of household wealth index overall and by community in South Africa.
**Figure S3**. Household wealth score by educational attainment for individuals in Zambia.
**Figure S4**. Household wealth score by educational attainment for individuals in South Africa.
**Table S1**. By country, frequency of asset ownership, and the weights assigned to them in construction of household wealth index.
**Table S2**. Population attributable fractions of prevalent TB for household wealth and educational attainment in both countries.
**Table S3**. The adjusted associations between HIV status and measures of socioeconomic position.
**Table S4**. The adjusted associations, in Zambia, between measures of socio‐economic position and putative mediating factors.
**Table S5**. The adjusted associations, in South Africa, between measures of socio‐economic position and putative mediating factors.
**Table S6**. The adjusted associations between putative mediators and prevalent TB in Zambia.
**Table S7**. The adjusted assocations between putative mediators and prevalent TB in South Africa.

## Appendix 1 The wording of questions used to derive the mediating variables

### HIV status

Have you been tested for HIV before? Are you willing to disclose the result of that test?
[If yes continue…] What was the result? Negative Positive

### Household crowding

How many people – including children – live in your household? How many sleeping rooms does your household have?

### Indoor air pollution

What type of fuel does your household mainly use to keep warm inside the house during winter? (check only one option) Nothing Electricity Liquefied Petroleum Gas Kerosene/Paraffin Charcoal Wood Other
What type of fuel does your household mainly use for cooking? (check only one option) No cooking is done Electricity Gas Paraffin Charcoal Wood Other
[if charcoal or wood] What type of stove is usually used for cooking? Open fire Surrounded fire Stove with combustion chamber
Where does cooking mainly happen? (check only one option) Indoors in main house Indoors in separate building Outdoors

### Smoking

Have you ever smoked?
If you have stopped smoking, how old were you when you stopped? (if the participant has not stopped smoking record as…)

### Malnutrition

Did your household have to rely on any of the following in the last 18 months?… …Reducing number of meals or food intake
‘During the past three months, did it happen even once that you or any member of your family experienced hunger because you did not have any food to eat?’

### Diabetes

Have you ever been told you have diabetes?

### Alcohol consumption

How would you classify your drinking habits? Have never drunk Daily drinker Occasional drinker Ex‐drinker

### Migration

How many years have you lived in this community? Write down actual number, zero if less than one year…
