# Supplementary material for: Socio‐economic gradients in prevalent tuberculosis in Zambia and the Western Cape of South Africa
Source: Trop Med Int Health. 2018 Mar 24;23(4):375–90. doi: 10.1111/tmi.13038 (PMC6022780; doi:10.1111/tmi.13038)
Supplement: Supplementary file 1 — Figure S1. The distribution of household wealth index overall and by community in Zambia. Figure S2. The distribution of household wealth index overall and by community in South Africa. Figure S3. Household wealth score by educational attainment for individuals in Zambia. Figure S4. Household wealth score by educational attainment for individuals in South Africa. Table S1. By country, frequency of asset ownership, and the weights assigned to them in construction of household wealth index. Table S2. Population attributable fractions of prevalent TB for household wealth and educational attainment in both countries. Table S3. The adjusted associations between HIV status and measures of socioeconomic position. Table S4. The adjusted associations, in Zambia, between measures of socio‐economic position and putative mediating factors. Table S5. The adjusted associations, in South Africa, between measures of socio‐economic position and putative mediating factors. Table S6. The adjusted associations between putative mediators and prevalent TB in Zambia. Table S7. The adjusted assocations between putative mediators and prevalent TB in South Africa. [file TMI-23-375-s001.docx]

| **Zambia** | **Number of Individuals (%)** | **Weight** |  | **South Africa** | **Number of Individuals (%)** | **Weight** |
| --- | --- | --- | --- | --- | --- | --- |
| **Component** |  |  |  | **Component** |  |  |
| Housing type (ordered by *a* *priori* ranking) | | |  | Housing type (ordered by *a priori* ranking) | | |
| House, single dwelling | 32,351(56) | 0.0667 |  | House, single dwelling | 17,642(54) | 0.3408 |
| Cluster, multi-occupancy | 13,186(23) | 0.0154 |  | Cluster, multi-occupancy | 1,904(6) | 0.0077 |
| Flat | 7,838(14) | 0.0447 |  | Flat | 1,014(3) | 0.0290 |
| Traditional hut | 3,793(7) | -0.2236 |  | Traditional hut | 7,897(24) | -0.2743 |
| Servant quarters, caravan, tent, worker hostel or other | 641(1) | 0.0047 |  | Servant quarters | 1,474(5) | -0.1161 |
|  |  |  |  | Caravan, tent, worker hostel or other | 2,861(9) | -0.1253 |
| Flooring type (ordered by *a priori* ranking) | | |  | Flooring type (ordered by *a priori* ranking) | | |
| Wood, lino or tile | 1,233(2) | 0.0726 |  | Wood, lino or tile | 19,747(60) | 0.1862 |
| Cement | 47,958(83) | 0.2732 |  | Cement | 5,846(40) | 0.0018 |
| Dirt or other | 8,618(15) | -0.3179 |  | Dirt | 4,279(13) | -0.2253 |
|  |  |  |  | Other | 2,920(9) | -0.0561 |
| Toileting facilities (ordered by *a priori* ranking) | | |  | Toileting facilities (ordered by *a priori* ranking) | | |
| Private flush toilet | 13,371(23) | 0.3097 |  | Private flush toilet | 19,508(60) | 0.3874 |
| Shared flush toilet | 5,132(9) | 0.1206 |  | Shared flush toilet | 12,253(37) | -0.3556 |
| Latrine | 37,201(64) | -0.3437 |  | Latrine, open defecation, bucket, chemical toilet or other | 1,031(3) | -0.1040 |
| Open defecation, bucket, chemical toilet or other | 2,105(4) | -0.0014 |  |  |  |  |
| Source of drinking water (ordered by *a priori* ranking) | | |  | Source of drinking water (ordered by *a priori* ranking) | | |
| Piped to residence | 9,047(16) | 0.1265 |  | Piped to residence | 17,557(54) | 0.3587 |
| Piped to yard | 17,903(31) | 0.2610 |  | Piped to yard | 8,034(24) | -0.0969 |
| Public tap | 18,211(32) | -0.1688 |  | Public tap, inside well, traditional well or other | 7,201(22) | -0.3315 |
| Inside well or borehole | 6,820(12) | -0.0962 |  |  |  |  |
| Shallow well, traditional well, river or other | 5,828(10) | -0.1901 |  |  |  |  |
| Household assets (ordered by factor score) | | |  | Household assets (ordered as for Zambia) | | |
| Fridge | 11,709(34) | 0.3472 |  | Fridge | 23,192(77) | 0.2284 |
| Television | 19,992(58) | 0.3406 |  | Television | 25,460(85) | 0.2306 |
| Radio | 23,564(68) | 0.2138 |  | Radio | 23,716(79) | 0.1655 |
| Mobile telephone | 29,373(85) | 0.2119 |  | Mobile telephone | 25,085(84) | 0.0550 |
| Car | 2,509(7) | 0.1694 |  | Car | 4,575(15) | 0.1118 |
| Domestic worker | 1,385(4) | 0.1301 |  | Domestic worker | 762(3) | 0.0420 |
| Motorcycle | 332(1) | 0.0480 |  | Motorcycle | 779(3) | 0.0395 |
| Bicycle | 9,997(29) | -0.0255 |  | Bicycle | 2,582(9) | 0.0560 |

Supplemental Table 1. By country, frequency of asset ownership, and the weights assigned to them in construction of household wealth index.

Supplemental Figure 1. The distribution of household wealth index overall and by community in Zambia.

Supplemental Figure 2. The distribution of household wealth index overall and by community in South Africa.

Supplemental Figure 3. Household wealth score by educational attainment for individuals in Zambia.

Supplemental Figure 4. Household wealth score by educational attainment for individuals in South Africa.

Supplemental Table 2. Population attributable fractions of prevalent TB for household wealth and educational attainment in both countries.

| **Zambia** | | | |
| --- | --- | --- | --- |
|  |  | **Number of individuals (%)^2^** | **PAF (95%CI)^1, 2^** |
| Household asset score | Very low | 8042(24) | 23.5%(-10.7-47.1%) |
|  | Low | 10461(31) |  |
|  | Medium | 8600(25) |  |
|  | High | 6942(20) |  |
|  |  |  |  |
| Education completed | None | 1697(5) | 19.3%(-3.1-36.9%) |
|  | Primary | 10944(32) |  |
|  | Lower Secondary | 8763(26) |  |
|  | Upper Secondary | 9708(29) |  |
|  | University or College | 2933(9) |  |

| **South Africa** | | | |
| --- | --- | --- | --- |
|  |  | **Number of individuals (%)^3^** | **PAF (95%CI)^1, 3^** |
| Household asset score | Very low | 7616(25) | 13.5%(-0.6-25.6%) |
|  | Low | 7413(25) |  |
|  | Medium | 7445(25) |  |
|  | High | 7524(25) |  |
|  |  |  |  |
| Education completed | None | 1131(4) | 15.1%(7.7-21.9%) |
|  | Primary | 5705(19) |  |
|  | Lower Secondary | 5470(18) |  |
|  | Upper Secondary | 16411(55) |  |
|  | University or College | 1281(4) |  |

1. *Adjusted for age group, gender and community or region, with clustering by SEA accounted for using robust standard errors.*
2. *Excludes 401 individuals with missing age data.*
3. *Excludes 19 individuals with missing age data.*

Supplementary Table 3. The adjusted associations between HIV status ^1^ and measures of socioeconomic position.

|  | Zambia | | South Africa | |
| --- | --- | --- | --- | --- |
|  | Adjusted odds ratio (95% CI) ^2, 3^ | p value | Adjusted odds ratio (95% CI) ^2, 4^ | p value |
| Household wealth | | | | |
| Very low | Referent | <0.0001 | Referent | <0.0001 |
| Low | 1.03 (0.92-1.14) |  | 0.75 (0.65-0.87) |  |
| Medium | 0.85 (0.75-0.97) |  | 0.83 (0.71-0.97) |  |
| High | 0.60 (0.52-0.69) |  | 0.63 (0.53-0.75) |  |
|  |  |  |  |  |
| Individual educational attainment | | | | |
| None | 0.82 (0.69-0.98) | <0.0001 | 0.88 (0.68-1.13) | <0.0001 |
| Primary | Referent |  | Referent |  |
| Lower Secondary | 1.01 (0.93-1.11) |  | 0.84 (0.72-0.98) |  |
| Upper Secondary | 0.75 (0.68-0.84) |  | 0.66 (0.56-0.77) |  |
| College or University | 0.58 (0.50-0.68) |  | 0.28 (0.20-0.40) |  |

*1. The measure incorporating self-report (see Table 1).*

*2. Adjusted for age group, gender and community or region, with clustering by SEA accounted for using robust standard errors.*

*3. Excludes 401 individuals with missing age data.*

*4. Excludes 19 individuals with missing age data.*

Supplementary Table 4. The adjusted associations, in Zambia, between measures of socio-economic position and putative mediating factors.

|  | Adjusted odds ratio (95% CI)^1, 2^ | | | | | | | |
| --- | --- | --- | --- | --- | --- | --- | --- | --- |
|  | Smoking | Alcohol | Malnutrition | Diabetes | Indoor Air Pollution | Crowding | Migration | HIV ^3^ |
| Household wealth | | | | | | | | |
| Very low | Referent | Referent | Referent | Referent | Referent | Referent | Referent | Referent |
| Low | 0.77 (0.69-0.87) | 0.94 (0.87-1.02) | 0.91 (0.78-1.05) | 1.43 (1.12-1.83) | 0.73 (0.58-0.93) | 0.95 (0.86-1.06) | 1.23 (1.10-1.38) | 1.03 (0.92-1.14) |
| Medium | 0.61 (0.53-0.71) | 0.97 (0.88-1.07) | 0.56 (0.46-0.69) | 1.66 (1.27-2.17) | 0.28 (0.21-0.38) | 0.86 (0.77-0.97) | 1.47 (1.26-1.70) | 0.85 (0.75-0.97) |
| High | 0.55 (0.47-0.65) | 1.06 (0.94-1.19) | 0.36 (0.28-0.48) | 1.76 (1.21-2.56) | 0.12 (0.08-0.18) | 0.57 (0.49-0.65) | 1.95 (1.60-2.37) | 0.60 (0.52-0.69) |
| p value | <0.0001 | 0.11 | <0.0001 | 0.002 | <0.0001 | <0.0001 | <0.0001 | <0.0001 |
| Individual educational attainment | | | | | | | | |
| None | 1.06 (0.86-1.30) | 0.82 (0.70-0.95) | 0.98 (0.83-1.16) | 0.70 (0.52-0.94) | 0.98 (0.82-1.18) | 0.90 (0.79-1.03) | 1.35 (1.09-1.67) | 0.82 (0.69-0.98) |
| Primary | Referent | Referent | Referent | Referent | Referent | Referent | Referent | Referent |
| Lower Secondary | 0.75 (0.67-0.84) | 1.01 (0.94-1.08) | 0.84 (0.78-0.91) | 0.96 (0.79-1.15) | 0.63 (0.56-0.71) | 0.87 (0.82-0.93) | 1.11 (1.01-1.22) | 1.01 (0.93-1.11) |
| Upper Secondary | 0.58 (0.52-0.66) | 0.94 (0.86-1.02) | 0.65 (0.58-0.73) | 1.15 (0.88-1.51) | 0.43 (0.37-0.51) | 0.71 (0.66-0.77) | 1.49 (1.34-1.66) | 0.75 (0.68-0.84) |
| College or University | 0.56 (0.48-0.66) | 1.07 (0.95-1.20) | 0.50 (0.41-0.60) | 1.48 (1.09-1.99) | 0.20 (0.16-0.26) | 0.46 (0.40-0.52) | 2.38 (1.92-2.95) | 0.58 (0.50-0.68) |
| p value | <0.0001 | 0.006 | <0.0001 | 0.004 | <0.0001 | <0.0001 | <0.0001 | <0.0001 |

*1. Adjusted for age group, gender and community or region, with clustering by SEA accounted for using robust standard errors.*

*2. All models exclude 2410 Zambians with missing data on age, household crowding or migration.*

*3. The measure incorporating self-report (see Table 1).*

Supplementary Table 5. The adjusted associations, in South Africa, between measures of socio-economic position and putative mediating factors.

|  | Adjusted odds ratio (95% CI)^1, 2^ | | | | | | |
| --- | --- | --- | --- | --- | --- | --- | --- |
|  | Smoking | Alcohol | Malnutrition | Diabetes | Crowding | Migration | HIV ^3^ |
| Household wealth | | | | | | | |
| Very low | Referent | Referent | Referent | Referent | Referent | Referent | Referent |
| Low | 0.94 (0.83-1.06) | 1.17 (1.04-1.32) | 0.91 (0.68-1.22) | 1.38 (1.16-1.65) | 0.69 (0.54-0.87) | 0.34 (0.23-0.49) | 0.75 (0.65-0.87) |
| Medium | 0.80 (0.69-0.93) | 1.10 (0.96-1.26) | 0.85 (0.62-1.16) | 1.39 (1.12-1.71) | 0.71 (0.48-1.04) | 0.34 (0.23-0.50) | 0.83 (0.71-0.97) |
| High | 0.66 (0.56-0.76) | 0.88 (0.77-1.00) | 0.47 (0.32-0.67) | 1.52 (1.21-1.91) | 0.48 (0.33-0.70) | 0.25 (0.18-0.36) | 0.63 (0.53-0.75) |
| p value | <0.0001 | <0.0001 | <0.0001 | 0.0008 | <0.0001 | <0.0001 | <0.0001 |
| Individual educational attainment | | | | | | | |
| None | 0.84 (0.68-1.04) | 0.89 (0.74-1.07) | 0.91 (0.71-1.16) | 0.95 (0.77-1.17) | 0.96 (0.80-1.17) | 1.96 (1.35-2.84) | 0.88 (0.68-1.13) |
| Primary | Referent | Referent | Referent | Referent | Referent | Referent | Referent |
| Lower Secondary | 0.92 (0.83-1.03) | 0.94 (0.85-1.05) | 0.92 (0.81-1.04) | 1.17 (1.02-1.34) | 0.95 (0.86-1.05) | 0.81 (0.65-1.02) | 0.84 (0.72-0.98) |
| Upper Secondary | 0.52 (0.47-0.57) | 0.85 (0.78-0.93) | 0.83 (0.73-0.95) | 0.98 (0.85-1.12) | 0.75 (0.68-0.82) | 0.58 (0.46-0.74) | 0.66 (0.56-0.77) |
| College or University | 0.22 (0.18-0.28) | 0.77 (0.66-0.89) | 0.65 (0.51-0.83) | 1.45 (1.11-1.90) | 0.52 (0.42-0.64) | 0.42 (0.31-0.57) | 0.28 (0.20-0.40) |
| p value | <0.0001 | 0.001 | 0.009 | 0.0002 | <0.0001 | <0.0001 | <0.0001 |

*1. Adjusted for age group, gender and community or region, with clustering by SEA accounted for using robust standard errors.*

*2. All models exclude 961 South Africans with missing data on age, household crowding or migration.*

*3. The measure incorporating self-report (see Table 1).*

Supplementary Table 6. The adjusted associations between putative mediators and prevalent TB in Zambia.

| Putative Mediator | Adjusted OR for prevalent TB (95% CI) ^1, 2^ | p value |
| --- | --- | --- |
| Smoking | 2.27 (1.52-3.38) | 0.0001 |
| Alcohol | 1.92 (1.45-2.55) | <0.0001 |
| Malnutrition | 1.11 (0.80-1.55) | 0.53 |
| Diabetes | 1.31 (0.58-2.98) | 0.52 |
| Indoor Air Pollution | 1.50 (1.06-2.13) | 0.02 |
| Crowding | 0.95 (0.72-1.24) | 0.69 |
| Migration | 0.60 (0.42-0.85) | 0.004 |
| HIV ^3^ | 4.25 (3.14-5.75) | <0.0001 |

*1. Adjusted for age group, gender and community or region, with clustering by SEA accounted for using robust standard errors.*

*2. All models exclude 2410 Zambians with missing data on age, household crowding or migration.*

*3. The measure incorporating self-report (see Table 1).*

Supplementary Table 7. The adjusted assocations between putative mediators and prevalent TB in South Africa.

| Putative Mediator | Adjusted OR for prevalent TB (95% CI) ^1, 2^ | p value |
| --- | --- | --- |
| Smoking | 1.52 (1.26-1.84) | <0.0001 |
| Alcohol | 1.75 (1.50-2.03) | <0.0001 |
| Malnutrition | 1.18 (0.92-1.51) | 0.19 |
| Diabetes | 0.83 (0.62-1.11) | 0.20 |
| Crowding | 1.00 (0.82-1.22) | 0.99 |
| Migration | 0.82 (0.59-1.14) | 0.23 |
| HIV ^3^ | 2.76 (2.22-3.44) | <0.0001 |

*1. Adjusted for age group, gender and community or region, with clustering by SEA accounted for using robust standard errors.*

*2. All models exclude 961 South Africans with missing data on age, household crowding or migration.*

*3. The measure incorporating self-report (see Table 1).*
